# Chinese American Caregivers of Older Adults With Alzheimer’s Disease and Other Dementias: A Scoping Review

**DOI:** 10.1093/geroni/igaa057.520

**Published:** 2020-12-16

**Authors:** Mengyao Hu, Laura Grunin, Bei Wu

**Affiliations:** New York University, New York, New York, United States

## Abstract

With the rapid growth of aging populations, the number of older adults with dementia is increasing worldwide. While there is a significant amount of research on dementia caregivers, we know very little about Chinese American (the largest subgroup of the Asian American population in America) caregivers. Therefore, the aims of this study are to 1) conduct a scoping review by identifying existing studies on Chinese American dementia caregivers, 2) present the current state of the science on Chinese American dementia caregiving, and 3) provide direction for future research. Twenty-one studies were included in the final review with 3 main themes synthesized (care experience, utilization of programs/services, and recruitment for caregivers). Care experience included illness perception towards Alzheimer’s Disease and related dementia (ADRD) such as stigma and normalization of the disease process. Filial piety was another important cultural belief underpinning care experience. An underutilization of supportive programs/services among this population was identified. Additionally, the few existing programs/services for Chinese American caregivers as well as the barriers encountered when seeking these programs/services were seen in the literature. The strategies and barriers of the included research articles for recruitment of Chinese American caregivers are also discussed in this study. These findings provide an overview of the current knowledge about Chinese American caregivers and serve as a stepping stone for future studies on similar populations in promoting caregiver’s health and developing culturally sensitive caregiver support services.

